# Patient participation in the clinical pathway—Nurses’ perceptions of adults’ involvement in haemodialysis

**DOI:** 10.1002/nop2.241

**Published:** 2019-02-14

**Authors:** Tone E. Andersen‐Hollekim, Marit Kvangarsnes, Bodil J. Landstad, Bente A. Talseth‐Palmer, Torstein Hole

**Affiliations:** ^1^ Clinic of Medicine and Rehabilitation Møre and Romsdal Hospital Trust Ålesund Norway; ^2^ Faculty of Medicine and Health Sciences Norwegian University of Science and Technology Trondheim Norway; ^3^ Department of Health Sciences, Faculty of Medicine and Health Sciences Norwegian University of Science and Technology Ålesund Norway; ^4^ Research Unit Møre and Romsdal Hospital Trust Ålesund Norway; ^5^ Department of Health Sciences Mid Sweden University Östersund Sweden; ^6^ Levanger Hospital Nord‐Trøndelag Hospital Trust Levanger Norway; ^7^ Department of Clinical and Molecular Medicine, Faculty of Medicine and Health Sciences Norwegian University of Science and Technology Trondheim Norway; ^8^ School of Biomedical Science and Pharmacy, Faculty of Health and Medicine University of Newcastle and Hunter Medical Research Institute Newcastle Australia

**Keywords:** haemodialysis, next of kin, nurses, patient involvement, patient participation, shared decision‐making

## Abstract

**Aim:**

To develop knowledge of nurses’ perceptions of participation for patients treated with haemodialysis and their next of kin.

**Design:**

A qualitative study with a hermeneutic approach.

**Methods:**

The data were collected in 2015 through focus groups with 13 nurses in Central Norway.

**Results:**

The nurses reported that patient participation ranging from non‐involvement to shared decision‐making was related to whether dialysis was initiated as acute or scheduled. The restrictions required in chronic haemodialysis limited participation. The next of kin were not involved. The nurses highlighted interventions on both the individual and system levels to strengthen participation.

**Conclusion:**

Dialysis units should develop strategies for participation related to individual needs and design treatment in cooperation with patients and their families, ensuring involvement early in the clinical pathway. Further research is needed on issues related to next of kin, including their desired level of involvement.

## 1 INTRODUCTION

Chronic kidney disease (CKD) is a progressive, irreversible renal impairment (Jansen et al., 2013). The disease is divided into stages 1–5, where patients in stage 5 are dependent on dialysis treatment for symptom relief and survival (Jansen et al., 2013). Haemodialysis (HD) is the most common form of dialysis treatment worldwide (Ortiz et al., 2014; The Norwegian Directorate of Health, 2011). The treatment is rigorous and imposes physical and mental burdens on patients and their families (Saad et al., 2015). Comorbidities such as diabetes, complications of the cardiovascular system, loss of self‐esteem, anxiety, depression, sexual dysfunction and sleep disorders are common with CKD patients (Laudański, Nowak, & Niemczyk, 2013; Saad et al., 2015; The Norwegian Directorate of Health, 2011; Vazquez et al., 2003) and contribute to higher mortality and a poorer health‐related quality of life than that of the general population (Gerogianni et al., 2016; Hemmett & McIntyre, 2017; Jansen et al., 2013; Saad et al., 2015; Vazquez et al., 2003). Patient participation may improve symptom burdens such as anxiety and depression and provide patients with better treatment outcomes (Saad et al., 2015).

## 2 BACKGROUND

Patient’ rights have been formulated in several documents and guidelines worldwide, and in Norway, patient participation is imposed by law (The Patients' Rights Act, 2015; World Health Organization, 2013). The law indicates that patients are entitled to participate in the implementation of their health care and includes the right to participate in choosing between available and medically sound methods of examination and treatment (The Patients' Rights Act, 2015). Actively participating in decisions related to own health issues is an important element of self‐management in chronic diseases (Protheroe, Brooks, Chew‐Graham, Gardner, & Rogers, 2012). Patients who are involved in their own treatment are reported to be less anxious and depressed, are less vulnerable, show better adherence to treatment protocols and have more insight into their own disease (Algilani, James, & Kihlgren, 2016; Barello, Graffigna, & Vegni, 2012; Orsino, Cameron, Seidl, Mendelssohn, & Stewart, 2003; Sahlsten, Larsson, Sjöström, & Plos, 2008; World Health Organization, 2013).

However, there are several internationally identified challenges to participation, such as nurses’ attitudes and beliefs, insufficient training, differences in role expectations, context and illness severity (Aasen, Kvangarsnes, & Heggen, 2012, 2012; Longtin et al., 2010; Thompson, 2007). In a traditional patient role, patients are expected to be passive and “looked after” (Joseph‐Williams, Elwyn, & Edwards, 2014; Protheroe et al., 2012). These expectations may result in patients under‐communicating knowledge and desire to participate to not be perceived as a “difficult patient” (Frosch, May, Rendle, Tietbohl, & Elwyn, 2012). Low health literacy may prevent patients from participating, and some patients will have cultural backgrounds without traditions for autonomous decisions (Elwyn et al., 2012).

Prolonged illness experience was demonstrated to provide a greater desire for involvement; thus, a patient with a chronic illness is more likely to participate than a patient with an acute illness (Thompson, 2007). The patient–professional relationship is important and a greater trust in professionals gives the patient the confidence to allow health workers to act on his or her behalf. Trust often appears when the patient has little experience or knowledge or has serious illnesses. The patient's wish for involvement reflects a combination of these dimensions (Thompson, 2007). Potential barriers to patient participation were found to be modifiable by addressing attitudinal changes at the levels of the healthcare team, organization and patient (Joseph‐Williams et al. 2014). Eldh, Ekman, and Ehnfors (2006) showed that good conditions for patient participation occurred when information was based on individual needs and accompanied by explanations. Professionals should recognize each patient's unique knowledge and respect the individual's description of the situation, rather than just inviting the patient to participate in decision‐making (Eldh et al., 2006).

Earlier studies on participation in haemodialysis have mainly focused on older patients or on dialysis patients as a group regardless of age (Aasen, Kvangarsnes, & Heggen, 2012, 2012; Muthalagappan, Johansson, Kong, & Brown, 2013; Stryckers, Nagler, & Van Biesen, 2016; Tuso, 2013; Van Loon, Boereboom, Bots, Verhaar, & Hamaker, 2015). A study on participation from the perspectives of patients >75 years of age, next of kin and nurses suggested that participation was not well integrated in dialysis units and that both the elderly and their families struggled for their right to participate (Aasen, Kvangarsnes, & Heggen, 2012; Aasen, Kvangarsnes, & Heggen, 2012; Aasen, Kvangarsnes, Wold, & Heggen, 2011). Younger patients may have a greater interest in participating, possess more treatment knowledge and are more confident in decision‐making situations than older (Orsino et al., 2003; Yalamanchili et al., 2013).

In‐centre HD largely affects the lifestyle and family life of patients and the next of kin (Gerogianni et al., 2016). Patients on HD are dependent on treatment several days a week and are imposed with numerous restrictions that create a burden on everyday life (Gerogianni et al., 2016). Education, careers and family life may be put on hold, leading to a lower social and economic status, the development of psychological disorders and a lower quality of life (Gerogianni et al., 2016; Saad et al., 2015; Yalamanchili et al., 2013). The next of kin of patients with long‐term illness may perceive their role as a valuable part of being human but also a burden or an inevitable obligation (Liedstrom, Kihlgren, Skovdahl, & Windahl, 2014). The next of kin who perceived their role as a burden expressed feelings of isolation, anxiety and anger and were at risk of developing depression. These symptoms were more prominent in female spouses (Liedstrom et al., 2014). Ebadi, Sajadi, Moradian, & Akbari (2018) found that the next of kin of patients undergoing haemodialysis experienced unpredictable, uncontrollable stressors such as time conflicts between caregiving and occupational affairs, care‐induced fatigue and fear of the future. Aasen et al. (2011) showed that the next of kin of elderly patients on HD felt excluded and forgotten by health providers.

Nurses work closely with patients and, therefore, hold a key position in terms of patient participation (Coulter & Collins, 2011; Longtin et al., 2010; Thompson, 2007; Tobiano, Bucknall, Marshall, & Chaboyer, 2015). A close therapeutic relationship may be developed between nurses and patients on long‐term dialysis because they spend several hours a week together during treatment (Shahgholian & Yousefi, 2015). The dialysis nurses are responsible for treatment administration, information and guidance on topics such as fluids, diet and medication, among others. Nurses’ perceptions of patient participation are thus central.

Although several studies have been presented on patient participation in HD (Erlang, Nielsen, Hansen, & Finderup, 2015; Hemmett & McIntyre, 2017; Van Loon et al., 2015), we found no study regarding nurses’ perceptions of participation for patients aged 18–65 years through different phases of the clinical pathway. The current study adds new knowledge on nurses’ perceptions of patient participation for adults undergoing HD and their next of kin relationships, both in the initial and established phases of dialysis treatment. Adults and younger adults are likely to have needs and concerns that differ from those of older patients and lack of participation may have major consequences. The results from this study will provide knowledge to the field that may improve health care for ESRD/HD patients and their next of kin through adding a broader understanding of patient participation in different phases of the clinical pathway. The study posed the following question: how do nurses perceive participation for patients undergoing HD and their next of kin?

## 3 THE STUDY

### 3.1 Aim

We aimed to develop the knowledge of nurses’ perceptions of participation for patients treated with haemodialysis and their next of kin.

### 3.2 Design

The study was framed using a hermeneutic approach (Gadamer, 2010), focusing on how a new and holistic understanding is created from text through pre‐understanding and fusions of horizons within the hermeneutic circle.

### 3.3 Theoretical framework

We used Thompson’s (2007) framework to understand nurses’ perceptions of patient participation. The framework forms a base of patient‐desired involvement, with three elements important for understanding: components, levels and context. The components are described as contributions to action, participation in defining the problem, participation in the reflection process, participation in decision‐making and mutual emotional meetings. These components are connected to five levels of participation, ranging from non‐involvement to autonomous decision‐making. Patient participation is contextual, meaning patients may wish to be involved in some areas but not necessarily in others. The desire for participation may change over time, even in a similar context and the patient may move between the different levels.

### 3.4 Participants

We conducted a purposive sampling to answer the research question (Krueger & Casey, 2015). The inclusion criterion was registered nurses (RNs) working with patients on HD. Both experienced and less experienced Norwegian‐speaking nurses with different ages were included. Nurses with leadership roles were excluded because the power imbalance between leaders and the other participants may limit the dynamics in the focus groups. The units were small, comprising 5–16 nurses. Two of the units employed nephrologists and had an outpatient function and one unit was responsible for the education of patients and the next of kin through “kidney school,” initiation of acute dialysis and PD. Recruitment was carried out by the head nurses who communicated written information and consent forms to relevant informants. Twenty‐five RNs were invited to participate in the study: 15 accepted and 13 participated. Seven informants were kidney nurses or intensive care nurses with experience between 3 months to more than 30 years. All nurses were females. Each focus group consisted of four to five participants in accordance with recommendations (Krueger & Casey, 2015; Tong, Sainsbury, & Craig, 2007).

### 3.5 Data collection

The data were collected during the spring of 2015 through focus groups comprising 13 nurses employed in three different dialysis units in Central Norway. We considered focus groups to provide a wide range of information and insight through group discussions, where participants could state their points of view stimulated by interactions in the group (Krueger & Casey, 2015). Based on previous literature, the theoretical framework (Thompson, 2007) and the aim of the study, we developed a semi‐structured questioning route (Krueger & Casey, 2015), focusing on the nurses’ perceptions of participation for patients treated with haemodialysis and the next of kin (Table 1). The informants, the interviewer and an assistant were present during the focus groups (Krueger & Casey, 2015). The three sessions were audio recorded and lasted from 58–71 min. The assistant took field notes and summarized what had been said. The informants were given the opportunity to supplement. The recordings were transcribed verbatim by the first author. We experienced the research question to be thoroughly illuminated through the three focus groups. At the end of the third, no new information was provided, and we considered the data as saturated (Krueger & Casey, 2015).

**Table 1:** Questioning route

1. What happen when it is decided that the patient has to start on dialysis treatment?
2. What kind of information do you provide?
3. How are patients and next of kin involved in decision‐making regarding treatment choices?
4. How do you practice person‐centred care?
5. Which experiences do you have from home treatment?
6. What are your overall perceptions on patient participation in the initial phase?
7. How is the patient involved in their treatment?
8. What challenges do you experience in patient participation?
9. How can patient participation be strengthened?
10. Is there anything else you want to tell related to patient participation?

### 3.6 Ethical considerations

The study was approved by the Regional Committee for Medical and Health Research Ethics (REK 2014/1586) and approved by the Norwegian Data Inspectorate (case number 40336). Informed consent was obtained from all participants. The informants’ anonymity was ensured by giving informants the letters A, B, C, D and E and numbering the focus groups 1, 2 and 3.

### 3.7 Data analysis

We analysed the data using hermeneutics, which focus on interpretations of texts (Gadamer, 2010). The researchers interpreted the nurses’ perceptions of patient participation as expressed through focus groups by considering the structure of the transcribed text (Flick, 2014). The authors read the transcripts several times. Notes from the interactions between participants were emphasized (Krueger & Casey, 2015). The first reading was performed to form an overall impression of the text. In further reading, we aimed to grasp the informants’ world (Gadamer, 2010), looking beyond what is close at hand to develop a new understanding. We emphasized reading the text carefully, focusing on quotations and common and distinguishing features. The movement of understanding was constantly from the whole to part and back to the whole (Gadamer, 2010). The data were coded according to the patient participation in various phases of the clinical pathway and the nurses’ suggestions on how to strengthen participation. In the analysis, we considered Thompson’s (2007) components, levels and context. We then identified four themes and show an example of the development of one of the themes in Table 2. We emphasized confirming the themes through constantly comparing them with the transcripts (Krueger & Casey, 2015). The authors had several discussions of the findings and interpretations throughout the whole process before reaching a common understanding.

**Table 2:** Example of developing the first theme

Quotations	Subthemes	Theme
“They are just thrown into it, and do not know about the future” (A, group 1)	Acute treatment and lack of involvement	Between non‐involvement and shared decision‐making
“Being able to choose the right treatment requires time and continuous conversations. Ten minutes with a busy doctor answering phone calls at the same time is not enough” (D, group 1)	Information giving	
“We have this patient who is a fisherman... he connects to a night machine when he is at home sleeping. He is on the transplant waiting list, but is very happy with life as it is now.” (C, group 3)	To be in control	

### 3.8 Rigour

In qualitative studies, the presence of the researcher deeply influences the reality studied (Flick, 2014). The first author is an experienced dialysis nurse whose knowledge provided an understanding of the topics, field access and a sound basis for the development of an adequate questioning route (Krueger & Casey, 2015). However, the close field position caused pre‐established beliefs important to acknowledge and clarify (Wernet, 2014). A constructive outlook from co‐authors was important to develop an intersubjective understanding and assessment of the results. The use of focus groups provided rich data while evolving into engaging discussions, where comments triggered others to express their perceptions on the topic. We noticed that nurses with less experience expressed perspectives that somewhat differed from the experienced nurses, although no disagreements arose. We recorded the focus groups and took field notes and the participants verified the oral summary. The findings reflected what the participants said, and we used quotations to validate the themes (Krueger & Casey, 2015).

## 4 FINDINGS

Thirteen nurses from three local hospitals in Central Norway conveyed their perceptions of patient involvement in HD through focus groups. We identified the following themes: (a) between non‐involvement and shared decision‐making; (b) restricted self‐determination; (c) absent next of kin; and (d) the nurses’ role in shared decision‐making.

### 4.1 Between non‐involvement and shared decision‐making

The nurses experienced differences in involvement related to whether dialysis treatment was initiated acutely or was scheduled. The informants expressed that acute kidney failure required fast treatment initiation, implicating a vascular catheter and, thus, no time to discuss treatment options. The nurses mediated that patients entering the emergency room were severely ill and were often overwhelmed by the situation: “They are just thrown into it and do not know about the future” (A, group 1). The nurses conveyed it was difficult to involve patients who required acute dialysis. This indicates less involvement in acute situations. It was stated that patients with acute kidney failure had no actual treatment choices because patients initiated in HD tended to stick to this treatment throughout the course.

The nurses expressed that the situation was different for patients with scheduled dialysis. These patients were provided with much information during the initial phase and were expected to make decisions about in‐centre HD or home treatment. The decisions were initiated as patients approached dialysis by the nephrologist and/or the outpatient nurse. One nurse suggested this was not the optimal time: “Being able to choose the right treatment requires time and continuous conversations. Ten minutes with a busy doctor answering phone calls at the same time is not enough” (D, group 1).

Other obstacles to decision‐making were highlighted—that is, when the disease progressed to require acute HD treatment or when decision‐making failed to occur because patients did not initiate it themselves. The nurses reported how even CKD patients at the time of dialysis initiation could be too affected by the disease to make sound treatment decisions.

The nurses perceived that it could be difficult for patients to fully understand the dialysis modalities and this complicated their treatment decisions. Outpatients about to start on dialysis were invited to visit the HD unit and, if possible, the nurses arranged for a meeting between the new patient and a patient already on PD, a “PD ambassador.” However, the units had a low percentage of home treatment and the nurses reported how the number of patients on PD was decreasing. The nurses reflected on this and explained how the hospital could appear such as a haven, making patients choose in‐centre HD:

For a patient with no medical background, it is not so easy to see the choices equally. Outpatients get to meet the staff on a regular basis and may choose in‐centre HD because other patients have it and it feels like a safe solution. (A, group 2)

However, the nurses gave examples about patients who had decided on PD and felt satisfied with the treatment: “We have a patient who is a fisherman...he connects to a night machine when he is at home sleeping. He is on the transplant waiting list but is very happy with life as it is now.” (C, group 3).

This indicates that initial situations differ between non‐involvement and shared decision‐making.

### 4.2 Restricted self‐determination

When in‐centre HD was established, the nurses reported about how patients were required to follow a time‐consuming treatment schedule and were restricted on fluid and diet. HD was largely predetermined—typically, 4 hr three–four times a week. Additionally, the patients spent time on transportation to and from the hospital. The nurses expressed that the patients’ opportunities to influence treatment were limited to changing their days on dialysis and, to a certain degree, their hours of attendance:

They do need the dialysis. We cannot let them do everything they want, you know (….) That is a bit of a challenge (…) And if the doctor says you must have four hours then that is how it should be and most patients will accept it. (D, group 3)

Some nurses referred to patients requiring extra dialysis due to fluid overload or low clearance and reflected on how patients could be reluctant to increase treatment: “It is like a punishment, you know. Elsewhere in health care it is like; the more treatment the better. Here the extra treatment is a reminder of not being clever enough” (E, group 1). The nurses conveyed that the lack of adherence could be major problems among patients on HD and reported how they spent time repeating information on fluid and diet restrictions and medication. However, they experienced that patients often struggled to manage their restrictions, sometimes resulting in dangerous fluid overloads or potassium levels. According to the nurses, patients had problems processing the information provided: “We tell them over and over again, but still…they do not seem to remember much of what we say” (B, group 3). The nurses believed that patients who were involved in their own treatment would have a greater understanding of why they were subjected to restrictions. They welcomed patients’ interest in treatment, although it sometimes challenged the nursing role. However, the nurses experienced that patients on HD easily adopted a passive role.

### 4.3 Absent next of kin

According to the nurses, staff interaction with the patients’ next of kin was absent. The “kidney school” was mainly the only arena for nurses to meet with patients’ relatives. The nurses conveyed worries about the burden on the next of kin because dialysis treatment affected the whole family. They reported how spouses could be reluctant towards home treatment, worrying that the patients would not be able to manage it, thus create an extra burden on the spouses. The nurses expressed that they had tried to arrange for meetings with the next of kin and encouraged patients to bring their spouse or other family to consultations, without success:

It is astonishing that we do not see more of the next of kin. I am thinking of the spouses ... if my husband had been on dialysis three days a week, I would like to see what was happening. (B, group 1)

### 4.4 Nurses’ role in shared decision‐making

The nurses suggested strengthening participation by offering patients flexible hours for dialysis attendance, night‐time dialysis, a self‐care unit and home treatment and highlighted that their awareness of patient participation had to be raised: “I think we have to discuss it. Change the framework. We cannot do things the way we always have…We work quite traditionally. We are the nurses and they are the patients” (A, group 2).

They expressed their role to be well incorporated and difficult to abandon. One of the less experienced nurses conveyed that patients should experience participation from their very first meeting with the staff: “I think it is important. If not, they may easily feel that they are in a system where they do not have much to say in the matter… the doctors and nurses are the ones who decide” (A, group 3)*.* The nurses experienced a contradiction between what they considered important in patient treatment and what was possible to achieve due to provided resources: “Sometimes I feel that we work on assembly lines. There is no time for reflection. We just have to get through the day” (B, group 3).

## 5 DISCUSSION

The analysis showed how nurses perceived participation for dialysis patients and the next of kin. Participation varied between non‐involvement and shared decision‐making. In acute situations, the patients’ illness limited participation. The initial phase of chronic HD was characterized by information loads and treatment decisions and patients on in‐centre HD had their lifestyle limited by strict treatment protocols. The nurses experienced sparse contact with the patients’ next of kin and finally discussed their role in how to strengthen the involvement of patients and their families.

In Norway, the ability to choose between different treatment modalities is mandatory (The Patients' Rights Act, 2015). Dialysis treatment strongly affects the lives of the patients and their next of kin and it is important that they are involved in treatment decisions. Patients approaching dialysis were expected to make decisions about a preferred treatment, although this was not considered to be the optimal time for decision‐making. The nurses emphasized timing and ample time. This finding is in accordance with that of Tuso (2013) who claimed that shared decision‐making and discussion about “life with kidney disease” should occur among the patients, their families and healthcare team as early as CKD stage 4, in sufficient time before dialysis initiation. Poor timing may cause patients to rush into treatment without having had time to discuss the options (Morton, Tong, Howard, Snelling, & Webster, 2010).

The informants in the present study perceived that patients struggled to figure out which treatment was the most suitable. We argue that information about treatment itself may not be sufficient for new patients to imagine what effect the different treatments have on their everyday lives. Sound treatment solutions may be achieved when the patients’ values and preferences are considered and when health providers actively share their knowledge about treatment impact and outcome (Schatell & Alt Stec, 2008). Patients together with the next of kin should consider whether in‐centre or home treatment would be best suited according to their lifestyle. This situation requires dialogue and is consistent with Thompson’s (2007) components that are important for participation. However, the nurses expressed how treatment traditions influenced patients’ choices and made them choose in‐centre HD because this was the common and available treatment. Previous research has shown how treatments may be excluded due to in‐centre limitations, lack of information about the options or the physician's treatment preference (Morton et al., 2010; Young et al., 2012).

In our study, the nurses perceived patients on in‐centre HD as passive. This perception differs from previous research showing that younger patients are likely to participate (Orsino et al., 2003). Thompson (2007) describes the context as an important element for patient participation. HD units are technically oriented and dialysis nurses may appear as experts in the way they handle the dialysis machines and possess knowledge about advanced illness. This might create a distance towards patients and limit participation. The physical conditions and placing patients in a row during treatment may cause reluctance towards bringing up sensitive issues. In this context, patients may feel vulnerable and not in control and become passive (Larsson, Sahlsten, Segesten, & Plos, 2011). Dependency on scheduled treatment protocols to survive adds mental pressure on patients and may cause psychological problems such as anxiety and depression (Theofilou, 2011).

The nurses in our study perceived themselves in traditional nursing roles where the nurses are the experts who actively take care of, or treat, whereas the patients passively receive treatment and this is consistent with previous study findings (Barnes, Hancock, & Dainton, 2013; Longtin et al., 2010). Although the nurses valued more active patients, they also conveyed that patients who wanted involvement could challenge the nurses’ professional judgements, or undermine their competences. Previous research has shown that health providers worry about how patient involvement might make patients decide too much, although patients emphasized the value of making decisions jointly (Solbjør, By Rise, Westerlund, & Steinsbekk, 2011). Our findings indicate that a consensus does not exist concerning patient participation in the dialysis units studied. Some nurses conveyed their concerns about involving patients, while others reported how the nurses worked in traditional nursing roles. We argue that patient involvement should be rooted in clinic management and not being solely dependent on individual nursing preferences.

Dialysis nurses may develop close bonds to their long‐term primary patients and hereby feel a personal responsibility for patients’ adherence to treatment. When experiencing a mismatch between the role expectations—that is, when patients do not conform to treatment protocols—‐conflict may arise between respecting patients’ autonomous rights and nurses’ mandatory health‐promoting nursing practice (International Council of Nurses, 2012). This may result in a controlling behaviour. The nurses’ perceptions of themselves as “the nurses” and patients as “the patients” exhibit an “us” against “them” thinking (Meulen, 2015), which creates distance and obstacles to involvement. Additionally, this concept fits with traditional roles as nurses‐as‐experts and passive patients adhering to the nurses’ advices. If patients do not adhere, they are seen as lacking insight into what is best for them (Solbjør et al., 2011) and nurses may feel it necessary to correct this. According to Thompson (2007), providing information is not equal to patient involvement. When nurses provide information, they are facilitating participation at a low level. Transferring knowledge to patients through providing information remains an important part of nurses’ tasks. However, to facilitate involvement, nurses must additionally engage in dialogue, allowing patients themselves to define their needs (Thompson, 2007).

Our study showed that nurses experienced sparse contact with the patients’ next of kin. Previous research has revealed how long‐term illness imposes a heavy psychological burden on the patients’ families (Ebadi et al., 2018; Liedstrom et al., 2014). The next of kin may be forced to adjust their life to the patients’ scheduled treatments, neglecting themselves and constantly having to cope with a sense of unfulfilled tasks and worries about the future (Ebadi et al., 2018). The nurses in our study invited patients’ families to the units, without success. In our interpretation, this may indicate how health providers determine what is important, without consulting those concerned. We suggest that Thompson's (2007) framework focusing on components may also be applied to the next of kin. Ebadi et al. (2018) called for improved interaction among professional caregivers to understand the conditions of the next of kin, thereby improving the quality of life for both patients and their families.

The nurses in this study suggested several options to strengthen patient participation, including the willingness among nurses to abandon their traditional roles and involve patients at a higher level (Thompson, 2007). However, nurses are part of the healthcare team and should not be solely responsible for patient participation. A clear leadership is a key to developing understanding and acceptance for departmental changes (Rokstad, Vatne, Engedal, & Selbæk, 2015), and the overall responsibility for implementation of patient participation lies mandatory in the management (Health Authorities & Health Trusts Act, 2013; The Patients' Rights Act, 2015).

The nurses in the current study experienced an imbalance between tasks and resources provided. This may cause patient participation to be of less priority, as supported by the Eurobarometer Qualitative Study (2012) where the time aspect was emphasized. There is a general agreement that the growing demands and expectations towards health care are placing extra pressure on limited resources (Légaré, Ratté, Gravel, & Graham, 2008). Research has demonstrated a link between the work environment, including staff levels and patient outcomes (Prezerakos, Galanis, & Moisoglou, 2015; Rafferty et al., 2007). However, no robust evidence has been found, indicating more time is required on the engagement in patient participation than in usual clinical practice (Légaré et al., 2008). There is a need to discuss new ways of involving patients and the next of kin in participating in different phases in the clinical pathway.

### 5.1 Limitations

The current study presents nurses’ perceptions of patient participation and does not consider the patients’ own experiences. The nurses may have hesitated to express controversial views in front of focus group members, and different answers may have been provided in individual interviews. The units’ head nurses carried out the recruitment process. This may have affected the process; however, because the units were small, we proceeded to obtain as many informants as possible. This study has a qualitative design and our findings are not intended for generalization (Krueger & Casey, 2015; Polit & Beck, 2012). Our findings may still be applicable to other dialysis units.

## 6 CONCLUSION

Our study showed that nurses experienced challenges related to patient participation throughout the clinical pathway. Participation differed between non‐involvement and shared decision‐making, without next of kin involvement. Knowledge from the present study indicates that new approaches to patient participation are needed for HD patients. We suggest that dialysis units should accommodate the needs of patients where education, work and family life are particularly important and treatments should be designed individually in close cooperation with the patients and their families. This requires altering traditional nursing roles and involving patients more, implicating a clear leadership. Further research on how the next of kin would like to be involved in different phases of the clinical pathway is needed.

## CONFLICT OF INTEREST

No conflicts of interests have been declared by the authors.
